# Cellular and synaptic properties of molecularly defined neurons in the external globus pallidus

**DOI:** 10.1016/j.isci.2025.113002

**Published:** 2025-06-27

**Authors:** Linda M.C. Koene, Wilhelm Thunberg, Sten Grillner, Gilad Silberberg, Maya Ketzef

**Affiliations:** 1 Department of Neuroscience, Karolinska Institutet, 171 77 Stockholm, Sweden

**Keywords:** Physiology, Neuroscience, Cellular neuroscience, Cell biology

## Abstract

The external globus pallidus (GPe) is a central basal ganglia (BG) nucleus, involved in various sensorimotor functions. The two main types of GPe neurons, the prototypic and arkypallidal neurons, differ in developmental origin, axonal projections, and functional roles. Using *ex vivo* whole-cell patch-clamp recordings, we characterized the membrane properties, morphology, and synaptic connectivity of molecularly identified GPe neurons. Arkypallidal neurons had broader, slower action potentials with higher thresholds, and smaller somata, dendritic length, and surface area than prototypic neurons. Optogenetic activation of prototypic neurons inhibited both GPe subpopulations, but paired recordings revealed very sparse direct synaptic connectivity only between prototypic neurons. Moreover, parvalbumin-expressing prototypic neurons were not electrically coupled. This work provides a comprehensive description of the GPe microcircuitry and may provide insight into the functional role of the GPe within the BG network.

## Highlights

• Identified prototypic and arkypallidal neurons differ in their membrane properties
• Prototypic neurons have larger somata and surface area than arkypallidal neurons
• Sparse synaptic connectivity is found, and only between prototypic neurons
• No indication of electrical coupling between PV prototypic neurons

## Graphical abstract

**Figure:**
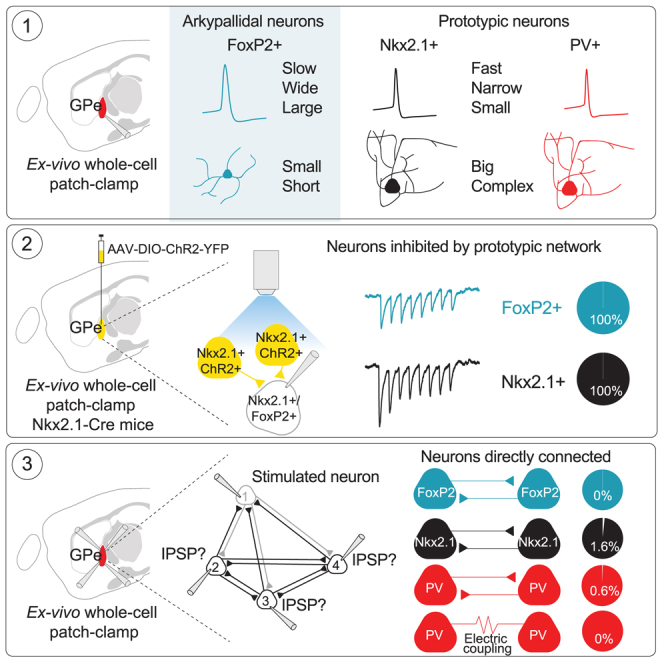

## Introduction

The external globus pallidus (GPe) is part of the basal ganglia (BG), a group of subcortical nuclei involved in a variety of sensorimotor functions and learning. In the classical view of the BG, the GPe is part of the indirect pathway.^1^^,^^2^ However, it is currently becoming clearer that the GPe is more than just a relay nucleus since it integrates inputs from multiple BG nuclei and projects to all of them.^3^^,^^4^^,^^5^^,^^6^^,^^7^^,^^8^^,^^9^^,^^10^^,^^11^^,^^12^^,^^13^

The GPe comprises two major subpopulations of GABAergic neurons: the prototypic and arkypallidal neurons.^3^^,^^9^^,^^14^^,^^15^^,^^16^ The prototypic neurons are the most prevalent, spiking at high frequencies, and projecting to various BG nuclei as well as thalamus and cortex.^17^ All prototypic neurons express Nk2 homeobox 1 (Nkx2.1) and subpopulations express parvalbumin (PV), and LIM homeobox protein 6 (Lhx6).^3^^,^^13^^,^^15^^,^^18^ Arkypallidal neurons are less prevalent and express forkhead protein 2 (FoxP2), neuronal PAS domain protein 1 (Npas1), and precursor preproenkephalin.^3^^,^^15^^,^^18^ These neurons are less active and project exclusively to the striatum.^16^ When active, the extensive axonal projections of these neurons deliver a strong and immediate inhibitory signal to medium spiny neurons (MSNs), suggesting them to be so-called “stop cells.”^19^ Prototypic and arkypallidal neurons also receive distinct inputs from other BG structures, shaping their responses to sensory stimulation.^20^

In addition to their afferent and efferent connectivity, GPe neurons also have collateral connections to neighboring neurons within the GPe. Previous anatomical and functional studies indicate that these connections are sparse, with a connectivity probability of 1%–1.4%.^21^^,^^22^ However, these studies were conducted in rats and focused on unidentified GPe neurons. Recent research from our laboratory, along with other studies, has shown optogenetically induced direct inhibition between prototypic neurons, between prototypic and arkypallidal neurons, but not between arkypallidal neurons in mice.^9^^,^^14^^,^^23^ Nevertheless, the synaptic connections within the GPe, the neuron-to-neuron organization rather than the network connectivity, remain unclear.

In addition to chemical synaptic connections, previous studies in the hippocampus,^24^^,^^25^ neocortex,^26^^,^^27^^,^^28^ and striatum^29^ have shown that PV neurons are electrically coupled through gap junctions, which play a crucial role in synchronous oscillatory activity. Histological reports have identified neuronal gap junctions in the GPe of humans^30^ and rats^31^; however, the specific cell types involved and their functional impact remain unclear. Given that increased synchronization of GPe neurons has been observed in animal models of Parkinson’s disease,^32^^,^^33^^,^^34^ electrical coupling between PV neurons may play an important role that has not been functionally studied yet.

Here, we aimed to obtain a detailed characterization of the cellular and synaptic properties of molecularly defined GPe neuronal subtypes. We first quantified the electrophysiological and morphological properties of prototypic (PV+ and Nkx2.1+) and arkypallidal neurons (FoxP2+) in mice. We then elucidated the synaptic and electrical connectivity between identified GPe neurons using multi-neuron patch-clamp recordings and optogenetic activation. Our results thus provide a comprehensive description of the functional organization of the GPe microcircuitry.

## Results

### Distinct electrophysiological properties reveal key differences between Nkx2.1+, PV+, and FoxP2+ neurons

**Figure 1:**
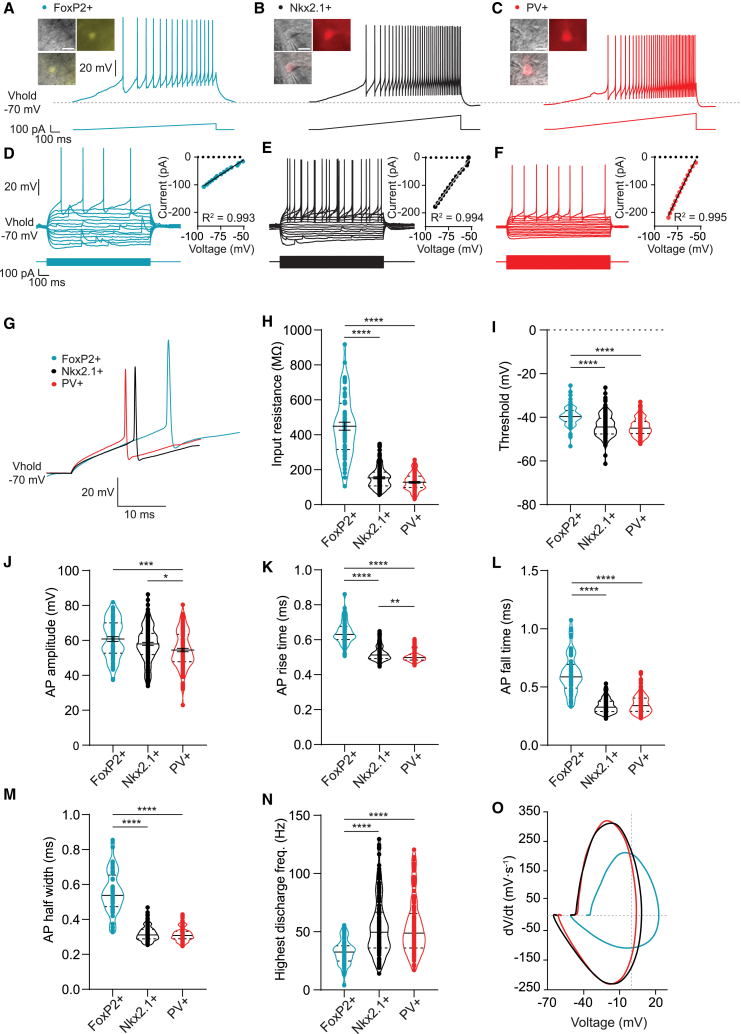
Electrophysiological properties of arkypallidal neurons are significantly different than prototypic neurons while minor differences are found between PV+ and Nkx2.1+ prototypic neurons (A) Representative voltage trace of an arkypallidal neuron in response to a current ramp injection. Insert: infrared, fluorescent, and merged images of an identified arkypallidal neuron (FoxP2+, YFP). The scale bars indicate 10 μm. (B) Representative voltage trace of a prototypic neuron in response to a current ramp injection. Insert: infrared, fluorescent, and merged images of an identified Nkx2.1 neuron (Nkx2.1+, TdTomato). The scale bars indicate 10 μm. (C) Representative voltage trace of a prototypic neuron in response to a current ramp injection. Insert: infrared, fluorescent, and merged images of an identified PV neuron (PV+, TdTomato). The scale bars indicate 10 μm. (D) Representative voltage trace of an arkypallidal neuron in response to increasing current steps. Insert: I-V curve based on the average voltage response at the end of the current step. A linear regression was fitted and is shown together with its R^2^ value. (E and F) Same as in (D) but then for a Nkx2.1+ neuron (E) and PV+ neuron (F). (G) Representative AP waveform of an arkypallidal, PV+ prototypic, and Nkx2.1+ prototypic neuron. (H–N) Violin plots of the electrophysiological properties of identified arkypallidal (FoxP2+), PV+ prototypic, and Nkx2.1+ prototypic neurons. The solid line shows the median or mean with SEM, and the upper and lower dashed lines indicate the quartiles. (O) Phase plot of a representative recording of all neuronal subtypes. Arkypallidal neuron (FoxP2+) in aqua, PV+ prototypic in red, and Nkx2.1+ prototypic neuron in black. When normally distributed, comparisons were made by a one-way ANOVA with Tukey’s *post hoc* multiple comparison test and otherwise by a Kruskal-Wallis ANOVA followed by Dunn’s multiple comparison test (FoxP2+: *n* = 66, 17 mice; Nkx2.1+: *n* = 150, 18 mice; PV+: *n* = 114, 13 mice). ∗*p* < 0.05, ∗∗*p* < 0.01, ∗∗∗*p* < 0.001, and ∗∗∗∗*p* < 0.0001. For more statistical details, see Table S1. Vhold, holding potential.

To study the electrophysiological properties and connectivity of GPe neurons, we obtained whole-cell recordings from prototypic neurons of Nkx2.1-cre or PV-cre mice crossed with an Ai9 tdTomato reporter mouse (hereafter referred to as Nkx2.1+ and PV+). Both Nkx2.1+ and PV+ tdTomato neurons are prototypic. PV+ neurons also express Nkx2.1 but Nkx2.1+ neurons can be both positive and negative for PV.^15^ To identify and patch the arkypallidal neurons, we used viral transduction to express a Cre-dependent fluorescent marker in FoxP2-cre mice (hereafter referred to as FoxP2+). We obtained detailed electrophysiological properties of FoxP2+ (*n* = 66), Nkx2.1+ (*n* = 150), and PV+ (*n* = 114) neurons. We applied a series of somatic current injections adjusted to the neurons’ membrane resistance, holding the neurons at −70 mV. For instance, a ramp protocol was used to calculate the action potential (AP) threshold and rheobase (see STAR Methods), and increasing current steps were used to calculate the input resistance and AP waveform properties (Figures 1A–1G). Our recordings show that arkypallidal neurons had a higher input resistance as well as lower threshold and rheobase (Figures 1H and 1I; Table S1 statistics). The calculated membrane capacitance (see STAR Methods) was significantly lower in the arkypallidal neurons than in the PV+ prototypic neurons (Table S1 statistics), and a similar trend was observed when compared with the Nkx2.1+ prototypic neurons (Table S1 statistics). APs in arkypallidal neurons showed higher amplitudes (Figure 1J), longer rise times (Figure 1K), longer fall times (Figure 1L), longer half-widths (Figure 1M) and duration, lower highest discharge frequency (Figure 1N), slower rise rates, slower fall rates, and longer afterhyperpolarization times than the Nkx2.1+ and PV+ prototypic neurons (Table S1 statistics). These differences are reflected in a phase plot made of representative neurons for each group (Figure 1O). The AP rise rate was not different between the arkypallidal and PV+ prototypic neurons but was significantly higher in Nkx2.1+ prototypic neurons, also when compared to PV+ prototypic neurons (Table S1 statistics). Additionally, we observed that the AP rise time of Nkx2.1+ prototypic neurons was significantly shorter and the AP amplitude significantly higher than in the PV+ prototypic neurons (Figure 1J; Table S1 statistics). Last, the amplitude of the fast afterhyperpolarization (fAHP) of the AP was significantly lower in PV+ prototypic compared to Nkx2.1+ prototypic neurons (Table S1 statistics). In sum, the most distinct differences were found between the arkypallidal and prototypic neurons; however, we also observed some differences between the PV+ and Nkx2.1+ prototypic neurons.

### Arkypallidal neurons have lower firing rates and higher sag ratios than prototypic neurons

**Figure 2:**
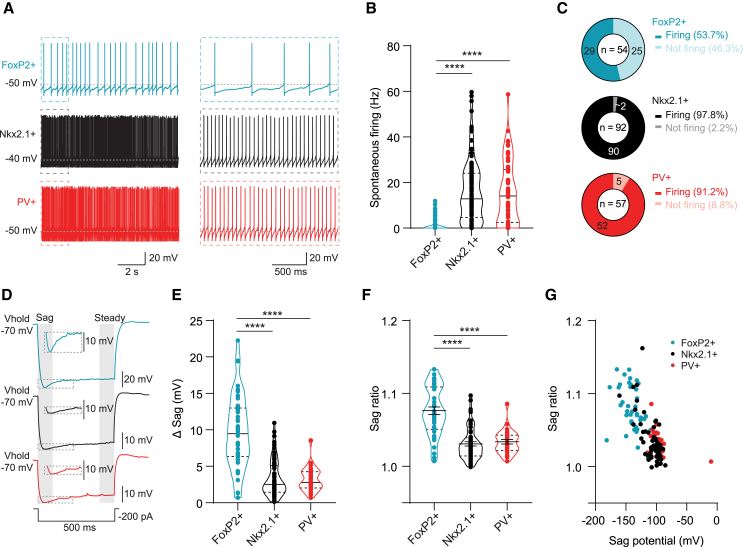
Arkypallidal neurons exhibit fewer spontaneous action potentials and have a higher sag ratio compared to prototypic neurons (A) Representative example traces of spontaneous action potential (AP) firing (no holding current) for arkypallidal neurons (aqua), PV+ prototypic (red), and Nkx2.1+ prototypic (black) neurons. The dotted square on the right is a magnified view of the dotted square shown on the left traces. (B) Violin plot quantification of spontaneous AP frequency with individual data points for each group. The solid line shows the median and the upper and lower dashed line indicate the quartiles. (C) Donut plots show the proportion of neurons firing and not firing (percentage in legend). (D) Representative example voltage response to a 200 pA hyperpolarizing current step showing a deeper sag in arkypallidal neurons. Note that the full trace has a different scale for the arkypallidal neuron. To highlight the difference, the inset shows the magnified part of the sag with the same scaling. The gray shading shows the sag and the steady state part of the trace that we used to calculate the sag ratio. (E) Violin plots with individual data points show significantly bigger sag potentials for arkypallidal neurons. The solid line shows the median and the upper and lower dashed line indicate the quartiles. (F) The sag ratio was calculated by dividing the voltage of the sag (gray field “sag”) by the steady-state voltage (gray field “steady”) and plotted in a violin plot with individual data points. The solid line shows the mean with SEM, and the upper and lower dashed lines indicate the quartiles (FoxP2+: *n* = 42, 14 mice; Nkx2.1+: *n* = 57, 13 mice; PV+: *n* = 28, 6 mice). Arkypallidal neurons have a significantly higher sag ratio. (G) Scatterplot of the sag potential, the lowest membrane potential where the sag is, vs. sag ratio, showing a separate cluster for arkypallidal neurons and the prototypic neurons. When normally distributed, comparisons were made by a one-way ANOVA with Tukey’s *post hoc* multiple comparison test and otherwise by a Kruskal-Wallis ANOVA followed by Dunn’s multiple comparison test. ∗∗∗∗*p* < 0.0001. For more statistical details, see Table S1.

To obtain a more detailed picture of the different neurons, we applied two additional protocols on a subset of neurons for each group (FoxP2+: *n* = 45, Nkx2.1+: *n* = 66, PV+: *n* = 28). First, we recorded the spontaneous firing rate when no holding current was injected. Evaluation of this rate (≥1 min) showed that arkypallidal neurons fire at a significantly lower rate compared to Nkx2.1+ and PV+ prototypic neurons (Figures 2A and 2B; Table S1 statistics). Moreover, a larger fraction of arkypallidal neurons were completely silent in comparison to the Nkx2.1+ and PV+ prototypic neurons (Figure 2C).

Second, we measured the sag and steady-state potential by injecting a 200 pA hyperpolarizing current while holding the neurons at −70 mV. We calculated the sag amplitude by subtracting the steady-state membrane potential from the most hyperpolarized point in response to the current step (Figure 2D). In line with previous reports,^23^^,^^35^ we observed a higher sag potential in arkypallidal neurons than in PV+ and Nkx2.1+ neurons (Figure 2E; Table S1 statistics). The sag ratio, calculated by dividing the sag potential by the steady potential, was significantly higher in arkypallidal neurons (Figure 2F; Table S1 statistics). The sag potential was similar in PV+ and Nkx2.1+ neurons but resulted in a separate cluster when plotted against the sag ratio in arkypallidal neurons (Figure 2G). Taken together, arkypallidal neurons fire less and show a higher sag ratio than prototypic neurons.

### Cluster analysis separates arkypallidal from PV+ and Nkx2.1+ prototypic neurons

**Figure 3:**
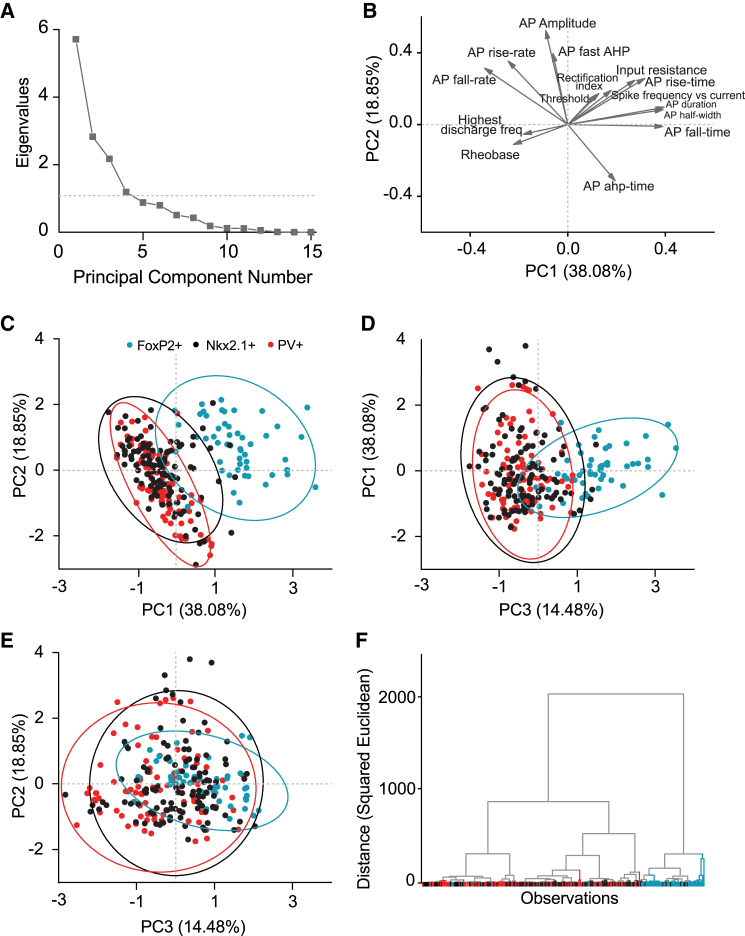
Unbiased clustering shows separate clusters for arkypallidal and prototypic neurons (A) Scree plot with the eigenvalues used to determine the number of principal components (PCs) that need to be used in the principal-component analysis (PCA). The gray dotted line is placed on eigenvalue 1. (B) Loading plot of the used active and passive membrane properties showing which parameters contribute most to the first two PCs. (C–E) Scatterplots with dots representing individual neurons for each group (FoxP2+ in aqua, PV+ in red, and Nkx2.1+ in black). The ellipses are visually supporting the different clusters (FoxP2+: *n* = 52, 17 mice; Nkx2.1+: *n* = 113, 18 mice; PV+: *n* = 89, 13 mice). (F) Dendrogram based on a hierarchical clustering analysis with Euclidean distance squared as the multi-dimensional distance metric and Ward’s method as the linkage rule, showing a separate cluster for arkypallidal neurons (aqua) and a mixed cluster representing both prototypic groups (PV+ in red and Nkx2.1+ in black).

To identify potential subtypes within arkypallidal (FoxP2+) and prototypic (PV+ and Nkx2.1+) neurons characterized by electrophysiological properties, we performed a principal-component analysis (PCA). We used 15 passive and active electrophysiological properties that included input resistance, AP amplitude, duration, half-width, rise-time, fall-time, afterhyperpolarization-time, rise-rate, fall rate, fAHP, rectification index, threshold, rheobase, highest discharge frequency, and spike frequency vs. current injection. We used four principal components based on the elbow bend in the eigenvalue plot (Figure 3A). The biplot visualizes how each parameter contributed to the PCA (Figure 3B). The PCA shows a clear cluster of arkypallidal neurons whereas the clusters of prototypic neurons, PV+ and Nkx2.1+, overlap (Figures 3C–3E). We observed a similar clustering pattern using a hierarchical clustering analysis showing a separate cluster for arkypallidal neurons and a mixed clustering of PV+ and Nkx2.1+ prototypic neurons (Figure 3F). Thus, despite having small differences between them, Nkx2.1+ and PV+ neurons still comprise the same group, separately from the arkypallidal neurons.

### Morphological differences between arkypallidal and prototypic neurons

**Figure 4:**
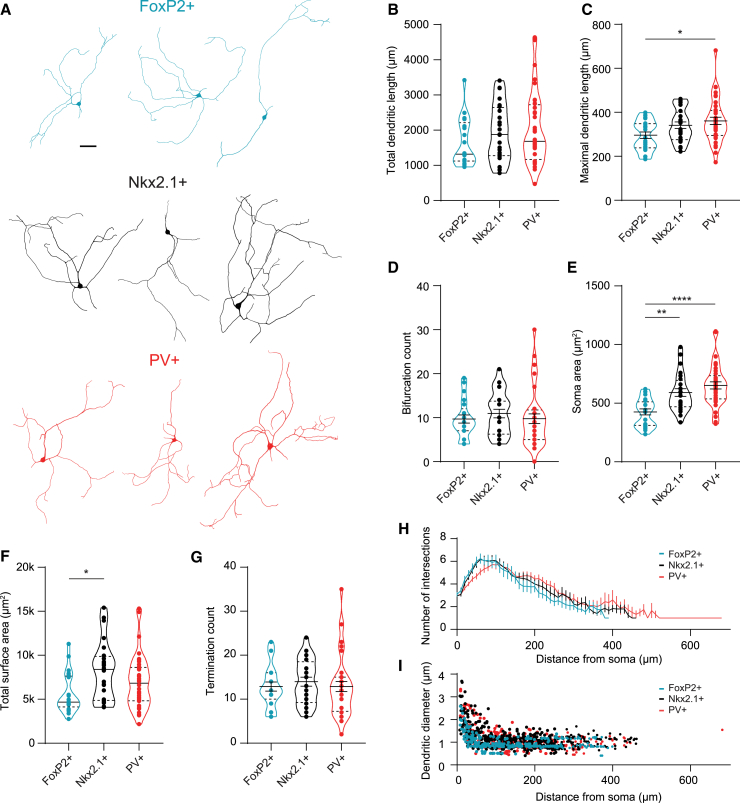
Arkypallidal neurons are smaller than prototypic neurons (A) Examples of reconstructed neurons for each group. The scale bars indicate 50 μm. (B–G) Quantification of the different morphological properties of arkypallidal and prototypic neurons. The solid line shows the median or mean with SEM, and the upper and lower dashed line indicates the quartiles. (H) Sholl analysis of the dendritic arborization of the arkypallidal and prototypic neurons (FoxP2+: *n* = 21; Nkx2.1+: *n* = 24; PV+: *n* = 36). The solid lines with SEM show the averages for each group. (I) Dendritic diameter plotted against distance from the soma (FoxP2+: *n* = 13; Nkx2.1+: *n* = 21; PV+: *n* = 11). When normally distributed, comparisons were made by a one-way ANOVA with Tukey’s *post hoc* multiple comparison test and otherwise by a Kruskal-Wallis ANOVA followed by Dunn’s multiple comparison test. ∗*p* < 0.05, ∗∗*p* < 0.01, ∗∗∗∗*p* < 0.0001. For more statistical details, see Table S2.

To allow morphological reconstructions, whole-cell patch-clamp recording pipettes were filled with neurobiotin. A total of 21 FoxP2+, 24 Nkx2.1+, and 36 PV+ neurons were successfully reconstructed and analyzed. Three representative reconstructions of somata and dendrites are shown in Figure 4A for each group. No differences were found in the total dendritic length (Figure 4B), bifurcation count (Figure 4D), termination count (Figure 4G), or number of primary dendrites between the GPe subpopulations (Table S2 statistics). However, arkypallidal neurons had shorter dendrites than PV+ prototypic neurons (Figure 4C; Table S2 statistics). Additionally, the total surface area of arkypallidal neurons was smaller than the Nkx2.1+ prototypic neurons (Figure 4F). The most significant difference was in the soma area, which was smaller in arkypallidal neurons compared to both the Nkx2.1+ and PV+ prototypic neurons (Figure 4E; Table S2 statistics). A Sholl analysis revealed that all groups had a maximum of 6 intersections and did not show differences between the different cell types (Figure 4H; Table S2 statistics). Last, we compared the dendritic diameters of 13 FoxP2+, 21 Nkx2.1+, and 11 PV+ neurons). The dendrites were sorted into 1^st^, 2^nd^, and >2^nd^ order dendrites and compared between groups. While the dendritic diameters were smaller in the distal regions in all groups, the arkypallidal neurons had significantly smaller dendritic diameters compared to both prototypic groups (Figure 4I; Table S2 statistics). Taken together, the arkypallidal neurons have smaller somata with narrower dendrites than prototypic neurons.

### Prototypic neurons provide GABAergic inhibition to both arkypallidal and prototypic neurons

**Figure 5:**
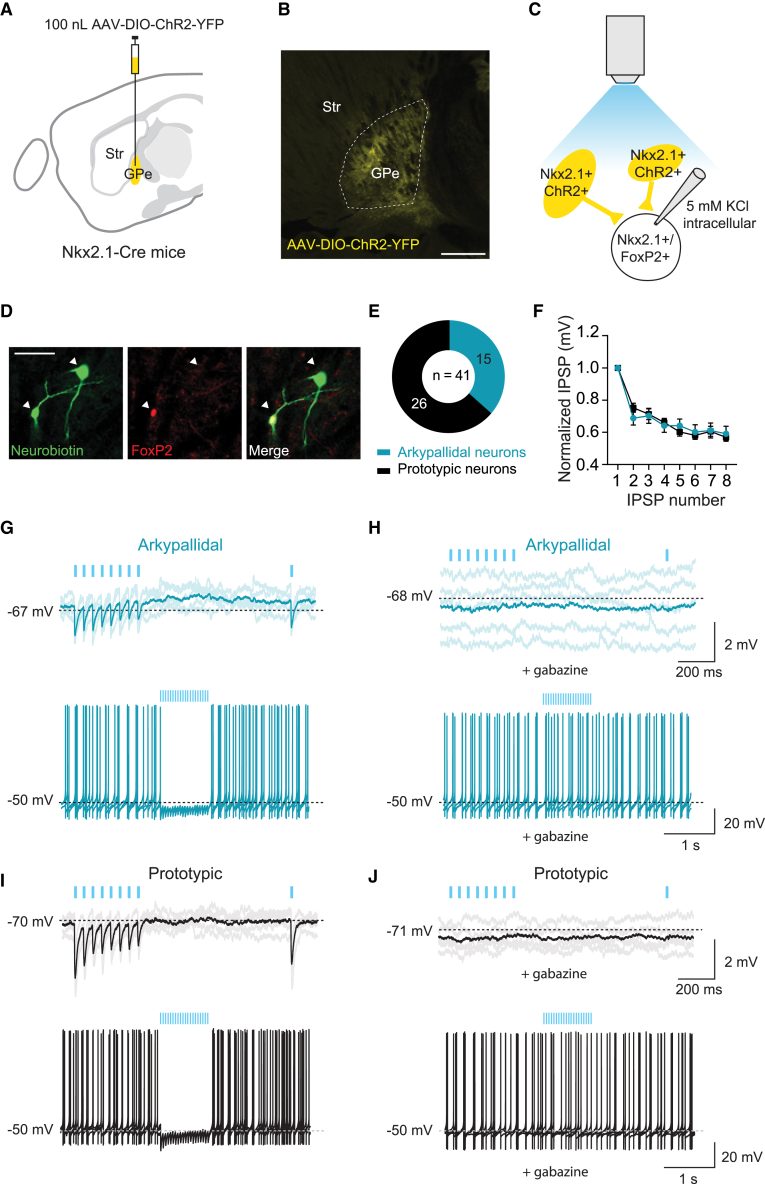
Prototypic network activity inhibits both neighboring arkypallidal and prototypic neurons (A) Schematic of AAV-DIO-ChR2-YFP virus injection. (B) Confocal image of the injection site in the GPe. The scale bars indicate 500 μm. (C) Schematic of the experimental setup. YFP-ChR2 positive neurons are stimulated while whole-cell recordings were made from the negative neurons within the GPe. (D) Example image of recorded neurons filled and stained with neurobiotin and a FoxP2 antibody. On the left is a FoxP2+ and on the right is a FoxP2− neuron highlighted by the white arrows. The scale bars indicate 50 μm. (E) Quantification of the number of neurons that were found FoxP2+ (arkypallidal neurons) and FoxP2− (prototypic neurons) (8 mice). (F) Normalized amplitudes of the responses to 20 Hz light stimulation of prototypic neurons recorded in neighboring prototypic (black, *n* = 23 neurons, 8 mice) and arkypallidal neurons (aqua, *n* = 12 neurons, from the same 8 mice). (G) Upper trace: representative average trace of an arkypallidal neuron upon a 20 Hz light stimulation train (8 pulses, 1 ms, 5 repetitions visualized in shadow) followed by a single light pulse (light blue). Lower trace: representative traces (3 overlapping) of a depolarized arkypallidal (+20 pA) neuron that gets inhibited by the prototypic network upon a 20 Hz light stimulation of 1 s. (H) The same arkypallidal neuron as in (G), following application of 10 μM gabazine, blocking the inhibition. (I and J) Same as in (G) and (H) but for a prototypic (FoxP2−) neuron.

Next, we studied the local connectivity within the GPe. Previous work has shown that prototypic neurons form collateral connections with surrounding prototypic and arkypallidal neurons whereas arkypallidal neurons do not.^9^^,^^14^ To have a closer examination of these connections between prototypic neurons in slices, we used a viral strategy to express fluorescently labeled channelrhodopsin (YFP-ChR2) in Nkx2.1+ prototypic neurons (Figures 5A and 5B). Neurons that did not express YFP, either arkypallidal neurons or prototypic neurons that were not transfected, were recorded while the network was optogenetically stimulated (Figure 5C). By *post hoc* FoxP2 immunostainings we identified the recorded neurons as FoxP2+, arkypallidal neurons (*n* = 15), and FoxP2−, prototypic neurons (*n* = 26) (Figure 5E). Both prototypic and arkypallidal neurons recorded in these conditions showed inhibition upon blue light illumination with similar levels of synaptic depression (Figures 5F, 5G, and 5I). The inhibitory postsynaptic potentials (IPSPs) in both groups were blocked upon gabazine application, showing that responses were mediated via GABA_A_ receptors (Figures 5H and 5J).

### Paired recordings reveal sparse synaptic connectivity between prototypic neurons

**Figure 6:**
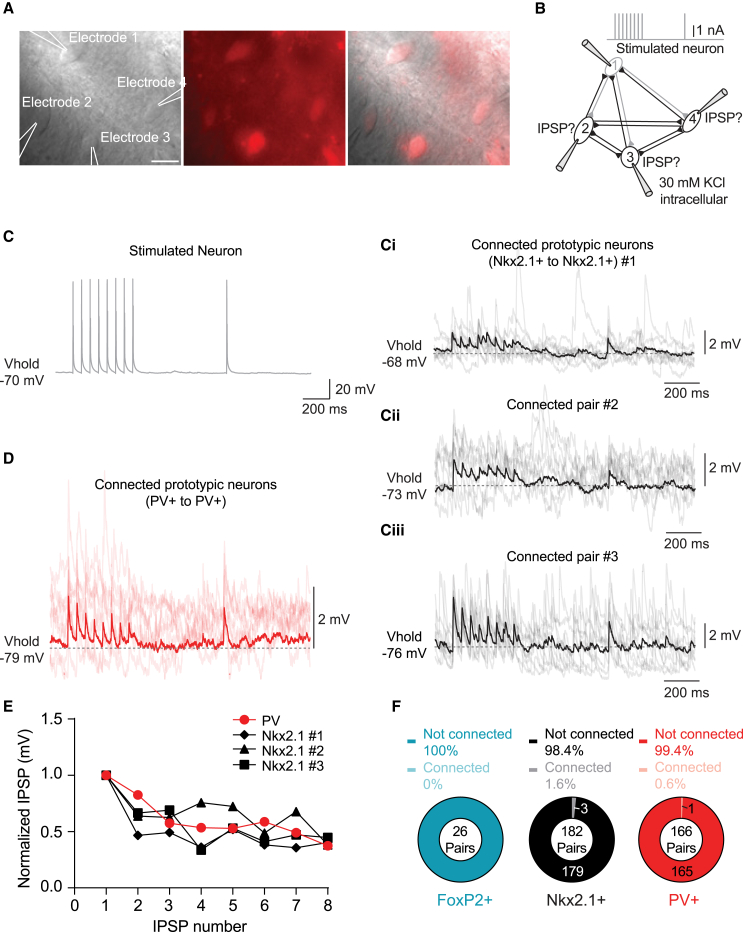
Sparse direct connectivity between prototypic neurons (A) Example infrared, TdTomato, and merged image of a quadruplet of identified Nkx2.1+ neurons. The scale bars indicate 10 μm. (B) Experimental schematic showing potential connections that can be studied when triggering APs in one neuron while observing potential IPSPs in the other neurons. In a quadruplet, up to 12 potential connections can be studied. (C) Upper left, an average example trace of a stimulated neuron (gray) showing a train of APs. (Ci, Cii, and Ciii) Average traces (black) and 8 individual sweeps in shading, of the Nkx2.1+ neurons that were unidirectionally connected with the Nkx2.1+ neuron. In total 3 connections out of 182 pairs were found (18 mice). Note that the scale bars in the traces are slightly different. (D) Average trace (red) and 8 individual sweeps in shading, of the only connection found between PV+ pairs (13 mice). (E) Normalized amplitudes of the IPSPs in response to 20 Hz stimulation from one prototypic neuron to the other. All connections show synaptic depression shown in (C and D). The Nkx2.1+ connections are depicted in black with different symbols representing the different connections and the PV+ connection is shown in red. (F) Donut plots summarizing the number of studied pairs in each group, the number of connections (number in the donut plot), and the percentage of connections found (in legend).

Optogenetic stimulation of prototypic neurons evoked GABAergic responses in all recorded GPe neurons; however, this approach did not provide information regarding the detailed connectivity between GPe neurons at the level of individual presynaptic and postsynaptic neurons. Therefore, we investigated the direct synaptic connections between identified prototypic and arkypallidal neurons using paired, triple, and quadruple patch-clamp recordings from neighboring GPe neurons (maximum distance between neuronal cell bodies 186 μm, average distance 80 ± 2.87 μm). We triggered trains of APs in each of the neurons while recording responses in the other neighboring neurons (Figures 6A and 6B). For the PV+ prototypic group, we used both sagittal and coronal slices to verify that the connectivity is not dependent on the cutting orientation as suggested in a previous study.^36^ Out of a total of 348 pairs of identified prototypic neurons (PV+ pairs *n* = 136 sagittal, 30 coronal; Nkx2.1+ pairs *n* = 182 sagittal), we found only 4 unidirectional connections: 3 connections between Nkx2.1+ neurons (1.7%, in sagittal slices; Figures 6C, 6E, and 6F) and 1 connection between PV+ neurons (0.6%, in sagittal slices; Figures 6D–6F). The distance between the somata of the Nkx2.1+ connected neurons was 99, 26, and 51 μm whereas for the PV+ connection, it was 76 μm. The amplitude of the first IPSP in the train varied between 1.2 and 3.7 mV. All 4 prototypic to prototypic connections showed synaptic depression (Figure 6E). Out of the 26 FoxP2+ arkypallidal to arkypallidal connections tested, none were connected (in sagittal slices; Figure 6F).

### Lack of synaptic connectivity between prototypic and arkypallidal neurons in paired recordings

To address the issue of connectivity between the groups, namely, prototypic to arkypallidal inhibition as seen using photostimulation, we included pairs of neurons in which one was fluorescently labeled and the other was negative. Based on their fluorescence and electrophysiological properties, pairs of putative arkypallidal and prototypic neurons were also tested for synaptic connectivity. In none of the recordings did we observe any connectivity between pairs of prototypic and arkypallidal neurons (total pairs 54, 20 pairs of FoxP2+/FoxP2− neurons; 30 pairs of Nkx2.1+/Nkx2.1− neurons; 4 pairs of PV+/PV− neurons). Thus, despite the widespread inhibition from prototypic neurons onto both prototypic and arkypallidal GPe neurons evoked by photostimulation, our data show that the direct synaptic connectivity between individual neighboring GPe neurons is extremely sparse.

### No evidence for electrical coupling between GPe PV-expressing prototypic neurons

**Figure 7:**
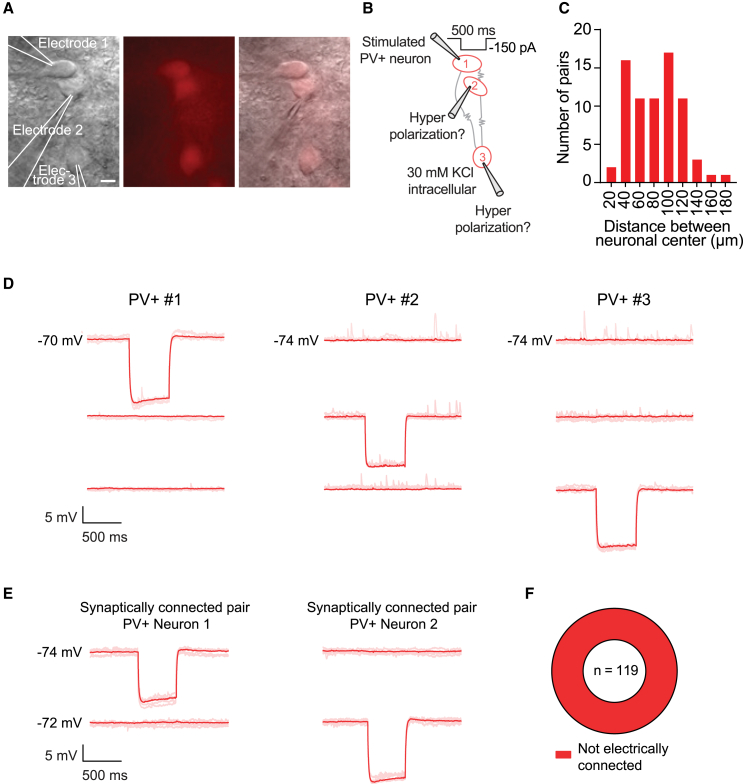
No evidence of electrical coupling between PV+ prototypic neurons (A) Example pictures (infrared, fluorescent, and merged) of a recorded triplet with PV+ neurons in proximity to each other. The scale bars indicate 10 μm. (B) Schematic of the experimental setup. Multi-electrode whole-cell patch clamp recordings of PV+ neurons were made and a −150 pA hyperpolarizing step of 500 ms was given to one of the neurons while observing the potential hyperpolarizing response in the other recorded neurons. (C) Bar graph showing the quantification of the distances between the recorded pairs measured from the center of the soma. (D) Example traces of the PV+ triplet are shown in (B). The average is shown in dark red with individual sweeps in shaded red. (E) Traces from the synaptically connected PV+ pair shown in Figure 6D, show no evidence of electrical coupling. (F) Donut plot showing none of the 119 neuron pairs tested for electrical coupling were connected (13 mice).

Since PV+ neurons in the neocortex, hippocampus, and striatum are electrically coupled via gap junctions,^24^^,^^25^^,^^26^^,^^27^^,^^28^^,^^29^ we also tested for electrical coupling between neighboring GPe PV+ neurons (Figure 7A). The experiments were performed in the same neurons as the membrane properties and synaptic connectivity. Due to the high KCl intracellular solution, inhibitory events were depolarizing at a holding potential of −70 mV, and a hyperpolarizing deflection was expected if the neurons were connected through gap junctions (Figure 7B). To ensure the presence of gap junctions was not overlooked due to the distance between the cell bodies, we recorded from neurons that were adjacent to each other (18 pairs >50 μm from each other measured from the center of the soma, Figures 7A–7C). Surprisingly, no electrically coupled neurons were observed in 119 pairs of PV+ prototypic neurons tested. This includes the PV+ pair that was synaptically connected as shown in Figure 6D (Figures 7D–7F).

## Discussion

Our study provides an extensive examination of electrophysiological and morphological characteristics together with synaptic connectivity of identified prototypic and arkypallidal neurons in the mouse GPe. First, we show that arkypallidal neurons have slower and larger action potentials, higher input resistance, lower spontaneous firing rates, and larger sag ratios. The differences between the PV+ and Nkx2.1+ prototypic neurons were more subtle, with only small differences in a few AP parameters. Second, we show that morphologically the arkypallidal neurons had smaller somata and dendritic trees. Third, we found that even though widefield photostimulation of Nkx2.1+ prototypic neurons inhibited all recorded neighboring neurons, the direct synaptic connections between individual GPe neurons were very sparse and were only found between prototypic neurons. Last, we did not observe electrical coupling between PV neurons.

When comparing the different electrophysiological characteristics of arkypallidal and prototypic neurons, most differences found were in line with previous reports.^18^^,^^23^^,^^35^^,^^37^ Interestingly, we identified subtle differences between the Nkx2.1+ and PV+ prototypic groups that have not been reported before. The AP of PV+ prototypic neurons have smaller amplitudes, shorter rise times, and slower rise rates than Nkx2.1+ prototypic neurons. Previous work in rats described differences within the prototypic neuron population suggesting there may be subtypes of prototypic neurons with potentially different functions.^18^ All PV+ neurons in the GPe express Nkx2.1; however, not all Nkx2.1+ neurons co-express PV, and can co-express Npas1 or Lhx6.^13^^,^^15^^,^^17^^,^^35^ Nonetheless, the degree of overlap in the expression of Lhx6 and PV in GPe neurons is unclear.^13^^,^^15^^,^^23^^,^^35^ It was also shown that PV+ neurons have different electrophysiological properties than Lhx6+ neurons.^13^ PV+ neurons in the GPe that project to different downstream nuclei have distinct membrane properties.^10^ These subpopulations are likely to contribute to the broad distribution of membrane properties within the PV+ and Nkx2.1+ neurons (Figures 1 and 2). Within the current study, we compared the PV+ neurons with Nkx2.1+ neurons and observed only small differences between these populations suggesting that both markers label largely overlapping prototypic subtypes.

In terms of somato-dendritic morphology, we observed a significantly smaller total surface area and soma size in arkypallidal neurons (Figure 4), which is in line with their lower capacitance (Table S1). Previous studies have shown differences in the axonal branching, in particular, the extensive axonal bifurcations of arkypallidal neurons in the striatum.^12^^,^^16^ These long-range axonal projections were cut in our slice experiments and were not quantified.

The functional implications of the electrophysiological and morphological differences between the GPe subpopulations are yet to be determined. For the prototypic population, high-frequency spiking, synaptic depression dynamics with small amplitude, sparse connectivity, and low input resistance can be linked to their role in inhibiting spontaneously active downstream nuclei (the subthalamic nucleus, internal globus pallidus, and substantia nigra pars reticulata). It is reasonable to speculate that small synaptic inputs to prototypic neurons will not affect their activity significantly because of these characteristics. Thus, prototypic neurons are more “tuned to their internal beat.” In contrast, the high input resistance of arkypallidal neurons makes them more tuned to synaptic inputs, which they should convey to inhibit MSNs of the striatum despite their low spontaneous spiking rate.

An important finding of this paper is the sparse local connectivity between GPe neurons. A recent study predicted, using an autocorrelation model on postsynaptic currents, that each PV+ prototypic neuron receives input from 1 to 2 other prototypic neurons.^36^ We found 1.15% of the prototypic-to-prototypic neurons studied to be directly synaptically connected. This number is close to the previously 1% predicted connectivity based on anatomy^21^ and the 1.4% reported based on electrophysiology.^22^ It should be noted that we tested the connectivity only between close neighbors and not between different areas within GPe and that the sparse connectivity may be an underestimation of the actual connectivity due to the elimination of connections due to slicing.

The functional role of such sparse connectivity between prototypic neurons remains unknown. The strength of the connections we and others found was considerable, indicating that one single neuron could influence the firing patterns of postsynaptic neurons.^22^ PV+ prototypic neurons project to the subthalamic nucleus as well as to striatal interneurons^38^ and arkypallidal neurons inhibit the striatum.^19^ Collateral inhibition in the GPe could, therefore, affect these BG structures via disinhibitory pathways. Additionally, if local connectivity in the GPe were higher, the nucleus might be less responsive to inputs from other BG nuclei due to reduced tonic activity, which would be undesirable for a central hub such as the GPe. How the local inhibition of prototypic neurons affects BG dynamics in health and disease requires further experimental as well as computational investigation such as was done for the striatum.^39^

A surprising result in our experiments is the absence of electrical coupling between PV+ neurons within the GPe, despite such coupling being reported in other brain areas^24^^,^^26^^,^^40^ and reports of neuronal gap junctions in postmortem human and rat GPe tissue.^30^^,^^31^ Even though we measured neurons that were adjacent to each other (<50 μm between somata of 18 pairs) and pipettes were loaded with neurobiotin, we did not observe electrical coupling nor dye coupling between GPe neurons. Interspecies differences could explain this discrepancy; for instance, strong electrical coupling was found in the hypothalamic dorsomedial arcuate nucleus of rats but was completely absent in mice.^41^ Unlike rats,^34^ there is little evidence of β-oscillations in mice^42^^,^^43^ (and unpublished own findings) suggesting that gap junctions may contribute to synchrony and β-oscillations in rats but not in mice. Additionally, gap junctions could be present in astrocytes rather than neurons since astrocytic gap junctions have been reported in Parkinsonian rats.^44^ Thus, our results suggest that gap junctions may not significantly influence GPe neuron activity in healthy mice, and their involvement in Parkinsonian mice remains to be determined.

To date, direct synaptic connections between arkypallidal neurons or arkypallidal to prototypic neurons have not been shown, either here or in other studies.^9^^,^^14^ Interestingly, arkypallidal neurons do have axon collaterals in GPe,^16^^,^^45^ which were computationally predicted to modestly inhibit other arkypallidal neurons.^46^ Inhibition of GPe neuronal subpopulations by prototypic neurons was observed in previous studies using optogenetic stimulation,^9^^,^^14^^,^^47^ suggesting that the intra-GPe synaptic organization may have specific spatial constraints other than inhibition of close neighbors as tested here.

In summary, we provide a detailed characterization of prototypic and arkypallidal neurons within the GPe. We show that arkypallidal neurons have distinct electrophysiological properties from both PV+ and Nkx2.1+ prototypic neurons. Between PV+ and Nkx2.1+ prototypic neurons, the differences were small and clustering analysis of electrophysiological properties did not support their classification as separate populations. In addition, even though network-wide optogenetic stimulation of prototypic neurons evoked inhibitory responses in all recorded GPe neurons, the direct synaptic connections between neighboring prototypic neurons were very sparse. Further work should be done to map the afferent and efferent connectivity of the GPe populations and to reveal their roles in BG functions and behavior.

### Limitations of the study

While our approach allows us to study the membrane properties and connectivity of molecularly identified GPe neurons, several limitations should be mentioned. First, slicing the brain allows us to optically identify neurons, however, it inevitably severs long-distance synaptic projections and inputs, depending on the orientation of cutting. To overcome this limitation, we recorded in slices that were cut at different orientations, yet this should be noted. Second, the use of transgenic mouse lines crossed with fluorescent reporter strains may also include positively labeled neurons that are only transiently expressing a molecular marker. To partially address this issue, we performed antibody staining and electrophysiological characterization.

## Resource availability

### Lead contact

Further information and requests for resources and reagents should be directed to and will be fulfilled by the lead contact, Maya Ketzef (maya.ketzef@ki.se).

### Materials availability

This study did not generate new unique reagents.

### Data and code availability

• All data reported (including the raw data) in this paper are available from the lead contact upon request.
• This paper does not contain new code. All codes used in this paper were previously published.^48^^,^^49^
• Any additional details required to study the data presented in this manuscript are available from the lead contact upon request.

## Acknowledgments

We thank Elin Dahlberg for technical support. We also thank Abdel El Manira and members of the Silberberg lab for comments on earlier versions of the manuscript. This work was supported by the Swedish Research Council (VR-M 2020-01785 to M.K. and VR-M 2023-02304 to G.S.), Karolinska Institutet Strategic Program for Neuroscience starting grant (StratNeuro to M.K.), grants from Karolinska Institutet, the Knut and Alice Wallenberg Foundation (KAW 2017.0273 to G.S.), the Swedish Brain Foundation (Hjärnfonden FO2018-0107 and FO2023-0230 to G.S., and FO2022-0122 and FO2023-0103 to M.K.), and ParkinsonFonden 1489/23 to M.K.

## Author contributions

L.M.C.K. designed, performed, and analyzed patch-clamp experiments and wrote the paper. W.T. analyzed data. S.G. wrote the paper. G.S. designed research and wrote the paper. M.K. designed and performed experiments, and wrote the paper.

## Declaration of interests

The authors declare no competing interests.

## STAR★Methods

### Key resources table

**Table:** 

REAGENT or RESOURCE	SOURCE	IDENTIFIER
**Antibodies**		

Cy2 or Cy5-conjugated streptavidin antibody	Jackson ImmunoResearch Laboratories	RRID: AB_2337246
Rabbit-FoxP2 primary antibody	Abcam	cat#ab16046; RRID: AB_2107107
anti-Rabbit Cy5 secondary antibody	Jackson ImmunoResearch Laboratories	RRID: AB_2340607

**Bacterial and virus strains**		

AAV5.EF1a.DIO-eYFP (plasmid #27056)	Addgene	RRID:Addgene_27056
AAV5.EF1.dflox.hChR2(H134R)-eYFP.WPRE.hGH (plasmid #20298)	Addgene	RRID:Addgene_20298

**Chemicals, peptides, and recombinant proteins**		

Isoflurane, Forene	AbbVie AB (Apoteket)	Cat#506949
Temgesic	Indivior Europe Limited (Apoteket)	Cat#521634

**Experimental models: Organisms/strains**		

PV-Cre (B6; 129P2-Pvalbtm1(cre)Arbr)	the Jackson Laboratory	RRID:IMSR_JAX:008069
Nkx2.1-Cre (C57BL/6J-Tg(Nkx2-1-cre)2Sand/J)	the Jackson Laboratory	RRID:IMSR_JAX:008661
FoxP2-Cre (B6.Cg-Foxp2tm1.1(cre/GFP)Rpa/J)	the Jackson Laboratory	RRID:IMSR_JAX:030541
Td-Tomato mouse (Ai9)	the Jackson Laboratory	RRID:IMSR_JAX:007909

**Software and algorithms**		

OriginLab	OriginLab Corporation	N/A
Data acquisition and analysis: Igor Pro 6.37	Wavemetrics	https://www.wavemetrics.com
GraphPad Prism (version 8.0.1), Boston, Massachusetts USA	N/A	www.graphpad.com
ZEN software (version 3.4)	ZEISS	www.zeiss.com

**Other**		

neuTube	Feng et al.^48^	N/A
Treem	Hjort et al.^49^	N/A

### Experimental model and study participant details

#### Animals

All animal procedures were performed following the national guidelines approved by the local ethics committee of Stockholm Norra Djurförsöketiska Nämnd (ethical permit N2022/2020 to GS) and in accordance with the European Communities Council Directive of November 24, 1986 (86/609/EEC). In all experimental groups, we used both male and female mice aged between 8 and 14 weeks. In total 17 Nkx2.1-TdTomato, 14 Nkx2.1-Cre, 12 PV-TdTomato, and 17 FoxP2-Cre mice were used. The animals were group-housed and under a 12-hour light/dark schedule, given *ad libitum* access to food and water. We identified the prototypic neurons with two reporter mouse lines: PV-TdTomato and Nkx2.1-TdTomato. These lines were generated by crossing a homozygous Td-Tomato mouse (‘Ai9’, stock JAX:007909, the Jackson Laboratory) with either a PV-Cre (B6; 129P2-Pvalbtm1(cre)Arbr, JAX:0088069, the Jackson Laboratory) or a Nkx2.1-Cre mouse (C57BL/6J-Tg(Nkx2-1-cre)2Sand/J, JAX:008661, the Jackson laboratory). We identified the arkypallidal neurons by injecting a Cre-dependent fluorescent virus into FoxP2-Cre mice (B6.Cg-Foxp2tm1.1(cre/GFP)Rpa/J, JAX:030541). Mice that were injected with a virus were at least 6 weeks old. We kept the Cre lines heterozygous on a wild-type C57BL/6J background (stock # 000664, the Jackson Laboratory).

### Method details

#### Virus injections

We anesthetized male or female Nkx2.1-Cre and FoxP2-Cre mice aged between 6 and 8 weeks with isoflurane (Animal Anesthesia System, VetEquip, USA) before placing them in a stereotaxic frame (Harvard Apparatus, Holliston, MA). We used a Quintessential Stereotaxic Injector (Stoelting, Wood Dale, IL) to inject 0.2 μL of AAV5.EF1a.DIO-eYFP (plasmid #27056) or 0.1 μL AAV5.EF1.dflox.hChR2(H134R)-eYFP.WPRE.hGH (plasmid #20298) virus in the GPe (from Bregma: AP -0.35 mm, ML +2.15 mm, DV -3.65 mm) at a rate of 0.1 μL/min. The injection pipette was kept in place for 5 minutes before it was slowly retracted. We gave the mice analgesics following the surgery (Buprenorphine, 0.08 mg/kg, i.p.).

#### Slice preparation and solutions

We used the virus-injected mice for *ex vivo* electrophysiological experiments 2–6 weeks after the injection. Mice were deeply anesthetized with isoflurane followed by decapitation. We quickly removed the brain and placed it in an ice-cold cutting solution containing (in mM): 205 sucrose, 10 glucose, 25 NaHCO3, 2.5 KCl, 1.25 NaH2PO4, 0.5 CaCl2, and 7.5 MgCl2. We prepared parasagittal or coronal slices of 250 μm containing the GPe with a Leica VT 1000S vibratome using the same cutting buffer saturated with 95% oxygen and 5% carbon dioxide. The slices were then transferred to a slice chamber filled with artificial cerebrospinal fluid (ACSF) saturated with 95% oxygen and 5% carbon dioxide and kept for 30 min at 34°C. The ACSF consisted of (in mM): 125 NaCl, 25 glucose, 25 NaHCO_3_, 2.5 KCl, 2 CaCl_2_, 1.25 NaH_2_PO_4_, and 1 MgCl_2_. After that, we moved the slices to room temperature and left them to recover for at least an hour before starting experiments.

Borosilicate pipettes with filament (Hilgenberg, Germany) of 7–8 MΩ resistance were pulled with a micropipette puller (P-1000, Sutter Instruments Co, Novato, CA). The intracellular solutions contained either (in mM) 105 K-gluconate and 30 KCl or 130 K-gluconate and 5 KCl with 10 HEPES, 4 Mg-ATP, 0.3 GTP, 10 Na_2_-phosphocreatine (pH ∼7.25, osmolarity ∼285 mOsm) and 0.3% neurobiotin (Vector laboratories, CA). To isolate and enhance inhibitory synaptic connections, we used a 30 mM KCl intracellular solution. Since we performed the experiments in series, the same intracellular was used for the membrane properties. The only exception was the optogenetic stimulation experiments where we used a 5 mM KCl intracellular solution (Figure 5).

#### Data acquisition

We visualized the neurons with infrared differential interference contrast (IR-DIC) microscopy (BX51WI, Olympus, Japan), a 40× long-working-distance immersion objective, and a digital camera (Hamamatsu Photonics, Japan). Fluorescent neurons were identified with an LED light source (CoolLED pE-300lite, United Kingdom). Both fluorescent as well as non-fluorescent neurons were whole-cell patch clamped in 35°C ACSF while saturated with 95% oxygen and 5% carbon dioxide. For the cell-to-cell connectivity experiments, we picked neurons within the same field of view having a maximum distance of 186 μm and an average of 160 μm between the neurons.

We conducted all experiments in the current clamp mode. Recordings were amplified using a MultiClamp 700B amplifiers (Molecular Devices, CA, USA), filtered at 2 kHz, and digitized at 10–20 kHz using ITC-18 (HEKA Elektronik, Instrutech, NY, USA). Data acquisition was done using custom-made protocols in Igor Pro (Wavemetrics, OR, USA). In all recordings, pipette the capacitance and access resistance were compensated, while the liquid junction potential (estimated ∼11 mV) was not.^50^ Data were rejected when the access resistance was higher than 30 MΩ.

Since most of the neurons in the GPe are spontaneously active, we held the neurons at ∼ -70 mV to obtain the most reliable readout of the electrophysiological parameters. The holding was released only during the spontaneous firing rate and the optogenetic Nkx2.1+ prototypic neuron network stimulation experiments.

#### Protocols and drug application

To obtain different electrophysiological properties of the neurons, we applied a series of hyperpolarizing and depolarizing current steps and ramps which were adjusted to the neurons’ input resistance. Measurements and calculations were made while holding the neurons at ∼ -70 mV, a potential where the neurons are not spontaneously firing. The analysis included: input resistance (IR), AP threshold, rheobase, current-voltage relation (IV), rectification magnitude, highest discharge frequency, and a variety of AP waveform properties.

We obtained the IV relationship from a series of subthreshold and suprathreshold current steps adjusted to the neuronal IR. The IR was determined by the regression slope of a linear fit to the steady-state (0.8–0.9 s of the current steps) average membrane potential response to increasing current injections starting from a holding potential of ∼ -70 mV (Figures 1D–1F). The highest discharge frequency was determined by the highest current injected with a step protocol where the step size was adjusted to the neuron’s IR. This frequency was the highest within the protocol but not the maximum the neuron could fire. We defined the AP threshold as the voltage at which the first derivative (dV/dt) of the membrane potential value crossed 10 mV·s^−1^ in response to a current ramp depolarizing the neuron. The rheobase was the minimal current step required to trigger an AP. The rectification index was calculated as the ratio between IR at the hyperpolarized potential and the holding potential.

The AP waveform included: amplitude (threshold to peak), duration (time from threshold to time of the same mV on the decaying phase), half-width (time from threshold to time where the amplitude is 50%), rise and fall times (time from threshold to peak, time from peak amplitude to the time where the decaying phase has the same voltage as the threshold), rise and fall rates (amplitude divided by rise time, amplitude divided by fall time), and the amplitude and time of the fast afterhyperpolarization (fAHP, deepest voltage and the time that happens starting from the defined fall time).

We obtained the spontaneous firing rate at the voltage where 0 pA was injected into the cell for 1.5 minutes over 3 sweeps of 30 s from which the average was taken. The sag potential was extracted from hyperpolarizing steps of −200 pA lasting 500 ms. We calculated the sag ratio by dividing the lowest voltage at the beginning of the current step (sag) by the steady-state (steady) voltage at the end of the current step (Figure 2D).

A phase plot was created for a representative recording for each group, where the first derivative (dV/dt) was plotted against the membrane potential.

We delivered the optogenetic stimulation through the 40× objective by using an LED light source (CoolLED pE-300lite, United Kingdom) at a wavelength of 465 nm (see Figure 5C). We gave eight light pulses of 1 ms at 20 Hz in 5 sweeps with 3 s of time interval between sweeps. To quantify the amplitude ratio, the IPSPs decaying phases were first fitted with a double-exponential function. Then their amplitudes were obtained by subtracting the decay of preceding IPSPs. To block GABAergic inhibitory currents, we applied 10 μM gabazine (SR-95531, Sigma-Aldrich).

For the multi-neuron direct synaptic connectivity experiments, trains of 20 Hz were injected into one neuron (2 nA, 30 ms) while recording the responses in the other neurons for 20 sweeps with a 5 s time interval between sweeps (see Figure 6B). An average of 20 repetitions was calculated and plotted. The amplitude quantification of the IPSPs was performed similarly to the optogenetic-triggered IPSPs, by fitting a double exponential.

To functionally test the presence of gap junctions between PV+ prototypic neurons, we applied a hyperpolarizing step of −150 pA to one neuron while observing the corresponding potential reflection in the other recorded neurons (see Figure 7A). The −150 pA was given from a holding potential of −70 mV for 500 ms. The average was taken from 20 sweeps.

#### Histology

After the *ex vivo* patch clamp experiments, we fixed the slices overnight in 4% paraformaldehyde solution at 4°C. The next day, we transferred the slices to PBS until further processing. To stain the neurobiotin, we washed the slices with PBS followed by the application of Cy2 or Cy5-conjugated streptavidin antibody in PBS 1X + Triton 0.6% and 10% Normal Donkey Serum for ∼3 h at room temperature or overnight at 4°C (1:1000, Jackson ImmunoResearch Laboratories, RRID: AB_2337246). Slices from the Nkx2.1-Cre animals injected with AAV5-DIO-ChR2-YFP were also stained with a Rabbit-FoxP2 primary antibody (1:500, cat#ab16046, Abcam) and an anti-Rabbit Cy5 secondary antibody (1:500, Jackson ImmunoResearch Laboratories, RRID: AB_2340607).

#### Microscopy and neuronal reconstructions

We imaged the stained slices with a confocal microscope (ZEISS LSM 800) at 20× magnification. We acquired Z-stacks and tile scans of the neurobiotin-filled neurons by using ZEN software (version 3.4, ZEISS). In some cases, colocalization of neurobiotin and FoxP2 was evaluated for cell identification.

To analyze the morphology of the different GPe subtypes, we used confocal Z-stacks to make reconstructions using neuTube and quantified them with Treem.^39^^,^^48^^,^^49^

### Quantification and statistical analysis

#### Statistical analysis

We analyzed and plotted the data using GraphPad Prism (version 8.0.1) and OriginPro (OriginLab Corporation, 2019b). The membrane properties data were tested for Gaussian distribution, and based on that, groups were compared with a two-sided one-way ANOVA (parametric) or a Kruskal-Wallis test (non-parametric). If the test was significant, we continued with either Tukey’s test or Dunn’s test as multiple comparisons *post hoc* tests. The Sholl analysis was analyzed with a Two-way ANOVA comparing simple effects within rows. When violin plots are shown, the middle solid lines indicate the median and the dotted upper and lower lines are the quartiles. If data were parametric, the middle line shows the average with bidirectional error bars representing the standard error of the mean (SEM). Statistical significance is defined as ∗*p* < 0.05, ∗∗*p* < 0.01, ∗∗∗*p* < 0.001, and ∗∗∗∗*p* < 0.0001. What test was used is mentioned in supplementary statistics Tables S1 and S2, and the figure legends.

For the clustering analysis, we used 15 passive and active electrophysiological properties and included: AP amplitude, duration, half-width, rise time, fall time, afterhyperpolarization-time, rise rate, fall rate, fast afterhyperpolarization, rectification index, threshold, input resistance, rheobase, maximum spike frequency and spike frequency vs. current injection. Due to some missing values, we removed some of the recordings before running a principal component analysis (PCA). We performed a PCA on 52 FoxP2+, 89 PV+, and 113 Nkx2.1+ neurons on all parameters. The parameters were normalized using z-scores (standardized to N(0,1)). The number of components was determined by the ‘elbow bend in the scree plot,^51^ eigenvalues were higher than 1 and explained ∼80% of the variance.

We then performed hierarchical clustering with Euclidean distance squared as the multi-dimensional distance metric and Ward’s method as the linkage rule.^52^
