# Supplementary material for: Cellular and synaptic properties of molecularly defined neurons in the external globus pallidus
Source: iScience. 2025 Jun 27;28(8):113002. doi: 10.1016/j.isci.2025.113002 (PMC12284287; doi:10.1016/j.isci.2025.113002)
Supplement: Document S1. Tables S1 and S2 [file mmc1.pdf]

iScience, Volume *28*

Supplemental information

Cellular and synaptic properties of molecularly defined neurons in the external globus pallidus

Linda M.C. Koene, Wilhelm Thunberg, Sten Grillner, Gilad Silberberg, and Maya Ketzef

Supplementary Table S1 (relates to Main Figures 1 and 2). Electrophysiological properties of prototypic (Nkx2.1+ and PV+) and arkypallidal (FoxP2+) neurons.

Parameter	Normally distributed	ANOVA/Kruskal-Wallis	*Post hoc* (Tukey's/Dunn's test)	Median (Mdn)/Mean (M) ± SEM
**Input Resistance (MΩ)**	Nkx2.1+ (n = 128)<br>No (p < 0.0001)<br>PV+ (n = 104)<br>Yes (p = 0.17)<br>FoxP2+ (n = 58)<br>Yes (p = 0.65)	F (2, 287) = 253; p < 0.0001	Nkx2.1+ vs PV+<br>P = 0.099<br>Nkx2.1+ vs FoxP2+<br>P < 0.0001<br>PV+ vs FoxP2+<br>P < 0.0001	Nkx2.1+<br>M 156.2 ± 5.62<br>PV+<br>M 131 ± 4.69<br>FoxP2+<br>M 449 ± 22.7
**Cm (pF)**	Nkx2.1+ (n = 107)<br>No (p = 0.002)<br>PV+ (n = 79)<br>Yes (p = 0.016)<br>FoxP2+ (n = 39)<br>No (p = 0.035)	H (2) = 7.438; p = 0.024	Nkx2.1+ vs PV+<br>P = 0.99<br>Nkx2.1+ vs FoxP2+<br>P = 0.078<br>PV+ vs FoxP2+<br>P = 0.022	Nkx2.1+<br>Mdn 80.14<br>PV+<br>Mdn 84.63<br>FoxP2+<br>Mdn 69.63
**AP amplitude (mV)**	Nkx2.1+ (n = 150)<br>Yes (p = 0.44)<br>PV+ (n = 114)<br>Yes (p = 0.41)<br>FoxP2+ (n = 66)<br>Yes (p = 0.15)	F (2, 327) = 8.495; p = 0.0003	Nkx2.1+ vs PV+<br>P = 0.02<br>Nkx2.1+ vs FoxP2+<br>P < 0.13<br>PV+ vs FoxP2+<br>P = 0.0002	Nkx2.1+<br>M 57.82 ± 0.85<br>PV+<br>M 54.28 ± 0.99<br>FoxP2+<br>M 60.85 ± 1.36
**AP rise time (ms)**	Nkx2.1+ (n = 142)<br>No (p < 0.0001)<br>PV+ (n = 99)<br>No (p < 0.0001)<br>FoxP2+ (n = 65)<br>No (p = 0.071)	H (2) = 133.3; p < 0.0001	Nkx2.1+ vs PV+<br>P = 0.027<br>Nkx2.1+ vs FoxP2+<br>P < 0.001<br>PV+ vs FoxP2+<br>P < 0.0001	Nkx2.1+<br>Mdn 0.51<br>PV+<br>Mdn 0.49<br>FoxP2+<br>Mdn 0.63
**AP fall time (ms)**	Nkx2.1+ (n = 139)<br>No (p < 0.0001)<br>PV+ (n = 112)<br>No (p < 0.0001)<br>FoxP2+ (n = 64)<br>No (p = 0.025)	H (2) = 124.8; p < 0.0001	Nkx2.1+ vs PV+<br>P = 0.88<br>Nkx2.1+ vs FoxP2+<br>P < 0.0001<br>PV+ vs FoxP2+<br>P < 0.0001	Nkx2.1+<br>Mdn 0.33<br>PV+<br>Mdn 0.34<br>FoxP2+<br>Mdn 0.59
**AP half-width (ms)**	Nkx2.1+ (n = 138)<br>No (p < 0.0001)<br>PV+ (n = 112)<br>No (p = 0.0003)<br>FoxP2+ (n = 64)<br>Yes (p = 0.2)	H (2) = 141; p < 0.0001	Nkx2.1+ vs PV+<br>P = 0.99<br>Nkx2.1+ vs FoxP2+<br>P < 0.0001<br>PV+ vs FoxP2+<br>P < 0.0001	Nkx2.1+<br>Mdn 0.31<br>PV+<br>Mdn 0.31<br>FoxP2+<br>Mdn 0.54
**Duration (ms)**	Nkx2.1+ (n = 137)<br>No (p < 0.0001)<br>PV+ (n = 104)<br>No (p = 0.011)<br>FoxP2+ (n = 63)<br>Yes (p = 0.043)	H (2) = 133.6; p < 0.0001	Nkx2.1+ vs PV+<br>P > 0.99<br>Nkx2.1+ vs FoxP2+<br>P < 0.0001<br>PV+ vs FoxP2+<br>P < 0.0001	Nkx2.1+<br>Mdn 0.86<br>PV+<br>Mdn 0.85<br>FoxP2+<br>Mdn 1.15

**AP Threshold (mV)**	Nkx2.1+ (n = 150)<br>No (p = 0.0124)<br>PV+ (n = 113)<br>No (p = 0.013)<br>FoxP2+ (n = 66)<br>Yes (p = 0.064)	H (2) = 44.9; p < 0.0001	Nkx2.1+ vs PV+<br>P = 0.94<br>Nkx2.1+ vs FoxP2+<br>P < 0.0001<br>PV+ vs FoxP2+<br>P < 0.0001	Nkx2.1+<br>Mdn -44.44<br>PV+<br>Mdn -44.99<br>FoxP2+<br>Mdn -39.66
**AP rise rate (mV/ms)**	Nkx2.1+ (n = 150)<br>Yes (p = 0.75)<br>PV+ (n = 114)<br>Yes (p = 0.18)<br>FoxP2+ (n = 66)<br>Yes (p = 0.67)	F (2, 327) = 9.94; p < 0.0001	Nkx2.1+ vs PV+<br>P = 0.021<br>Nkx2.1+ vs FoxP2+<br>P < 0.0001<br>PV+ vs FoxP2+<br>P = 0.13	Nkx2.1+<br>M 108.8 ± 1.69<br>PV+<br>M 101.8 ± 2.04<br>FoxP2+<br>M 95.43 ± 2.5
**AP fall rate (mV/ms)**	Nkx2.1+ (n = 150)<br>Yes (p = 0.1)<br>PV+ (n = 114)<br>Yes (p = 0.18)<br>FoxP2+ (n = 66)<br>No (p = 0.033)	F (2, 327) = 40.23; p < 0.0001	Nkx2.1+ vs PV+<br>P = 0.3<br>Nkx2.1+ vs FoxP2+<br>P < 0.0001<br>PV+ vs FoxP2+<br>P < 0.0001	Nkx2.1+<br>M 172.6 ± 4.51<br>PV+<br>M 163 ± 5.25<br>FoxP2+<br>M 104.6 ± 4.62
**Highest discharge frequency (Hz)**	Nkx2.1+ (n = 145)<br>No (p < 0.0001)<br>PV+ (n = 106)<br>No (p < 0.0001)<br>FoxP2+ (n = 64)<br>Yes (p = 0.63)	H (2) = 54.37; p < 0.0001	Nkx2.1+ vs PV+<br>P > 0.99<br>Nkx2.1+ vs FoxP2+<br>P < 0.0001<br>PV+ vs FoxP2+<br>P < 0.0001	Nkx2.1+<br>Mdn 49.5<br>PV+<br>Mdn 48.75<br>FoxP2+<br>Mdn 32.5
**Rheobase (pA)**	Nkx2.1+ (n = 128)<br>No (p = 0.0001)<br>PV+ (n = 102)<br>No (p < 0.0001)<br>FoxP2+ (n = 60)<br>Yes (p = 0.073)	H (2) = 77.28; p < 0.0001	Nkx2.1+ vs PV+<br>P = 0.79<br>Nkx.1+ vs FoxP2+<br>P < 0.0001<br>PV+ vs FoxP2+<br>P < 0.0001	Nkx2.1+<br>Mdn 79.61<br>PV+<br>Mdn 81.11<br>FoxP2+<br>Mdn 40.55
**fAHP time (ms)**	Nkx2.1+ (n = 136)<br>No (p < 0.0001)<br>PV+ (n = 96)<br>No (p < 0.0001)<br>FoxP2+ (n = 65)<br>Yes (p < 0.12)	H (2) = 70.49; p < 0.0001	Nkx2.1+ vs PV+<br>P > 0.99<br>Nkx2.1+ vs FoxP2+<br>P < 0.0001<br>PV+ vs FoxP2+<br>P < 0.0001	Nkx2.1+<br>Mdn 0.45<br>PV+<br>Mdn 0.45<br>FoxP2+<br>Mdn 0.79
**fAHP amplitude (mV)**	Nkx2.1+ (n = 146)<br>Yes (p = 0.26)<br>PV+ (n = 105)<br>Yes (p = 0.79)<br>FoxP2+ (n = 66)<br>Yes (p = 0.08)	F (2, 314) = 8.374; p < 0.0001	Nkx2.1+ vs PV+<br>P = 0.0002<br>Nkx2.1+ vs FoxP2+<br>P = 0.49<br>PV+ vs FoxP2+<br>P = 0.065	Nkx2.1+<br>M 17.9 ± 0.42<br>PV+<br>M 15.33 ± 0.48<br>FoxP2+<br>M 17.13 ± 0.64
**Rectification index for steady state**	Nkx2.1+ (n = 135)<br>Yes (p = 0.18)<br>PV+ (n = 86)<br>No (p = 0.0017)<br>FoxP2+ (n = 53)<br>Yes (p = 0.12)	F (2, 271) = 66.61; p < 0.0001	Nkx2.1+ vs PV+<br>P = 0.067<br>Nkx2.1+ vs FoxP2+<br>P < 0.0001<br>PV+ vs FoxP2+<br>P < 0.0001	Nkx2.1+<br>M 1.23 ± 0.020<br>PV+<br>M 1.35 ± 0.043<br>FoxP2+<br>M 1.93 ± 0.08
**Discharge vs current**	Nkx2.1+ (n = 132)<br>No (p < 0.0001)	H (2) = 57.04; p < 0.0001	Nkx2.1+ vs PV+<br>P = 0.071	Nkx2.1+<br>Mdn 0.33

	PV+ (n = 94)<br>Yes (p = 0.36)		Nkx2.1+ vs FoxP2+<br>P < 0.0001	PV+<br>Mdn 0.30
	FoxP2+ (n = 56)<br>No (p < 0.0001)		PV+ vs FoxP2+<br>P < 0.0001	FoxP2+<br>Med 0.58
**0 pA spontaneous frequency (Hz)**	Nkx2.1+ (n = 101)<br>No (p = 0.0007)	H (2) = 64.65; p < 0.0001	Nkx2.1+ vs FoxP2+<br>P < 0.0001	Nkx2.1+<br>Mdn 14.10
	PV+ (n = 57)<br>No (p < 0.0001)		Nkx2.1+ vs FoxP2+<br>P < 0.0001	PV+<br>Mdn 12.81
	FoxP2+ (n = 48)<br>No (p < 0.0001)		PV+ vs FoxP2+<br>P < 0.0001	FoxP2+<br>Mdn 0
**Sag ratio**	Nkx2.1+ (n = 57)<br>No (p = 0.0006)	F (2, 124) = 43.27; p < 0.0001	Nkx2.1+ vs PV+<br>P = 0.021	Nkx2.1+<br>M 1.03 ± 0.003
	PV+ (n = 28)<br>Yes (p = 0.08)		Nkx2.1+ vs FoxP2+<br>P < 0.0001	PV+<br>M 1.03 ± 0.003
	FoxP2+ (n = 42)<br>Yes (p = 0.29)		PV+ vs FoxP2+<br>P = 0.13	FoxP2+<br>M 1.08 ± 0.005
**Sag (mV)**	Nkx2.1+ (n = 66)<br>No (p < 0.0001)	H (2) = 52.55; p < 0.0001	Nkx2.1+ vs PV+<br>P > 0.99	Nkx2.1+<br>Mdn 2.78
	PV+ (n = 28)<br>No (p = 0.04)		Nkx2.1+ vs FoxP2+<br>P < 0.0001	PV+<br>Mdn 2.53
	FoxP2+ (n = 45)<br>Yes (p = 0.64)		PV+ vs FoxP2+<br>P < 0.0001	FoxP2+<br>Mdn 9.46

Supplementary Table S2 (relates to Main Figure 4). Morphological properties of prototypic (Nkx2.1+ and PV+) and arkypallidal (FoxP2+) neurons.

**Parameter**	**Normally distributed**	**ANOVA/Kruskal-Wallis**	***Post hoc* (Tukey's/Dunn's test)**	**Median (Mdn)/Mean (M) ± SEM**
**Termination count**	Nkx2.1+ (n = 24)<br>Yes (p = 0.93)	F (2, 78) = 0.29; p = 0.74	Nkx2.1+ vs PV+	Nkx2.1+<br>M 13.22 ± 1.018
	PV+ (n = 36)<br>Yes (p = 0.51)		Nkx2.1+ vs FoxP2+	PV+<br>M 12.81 ± 1.168
	FoxP2+ (n = 21)<br>No (p < 0.001)		PV+ vs FoxP2+	FoxP2+<br>M 12.81 ± 1.046
**Total dendritic length (µm)**	Nkx2.1+ (n = 24)<br>No (p = 0.0105)	H (2) = 2.32; p = 0.31	Nkx2.1+ vs PV+	Nkx2.1+<br>Mdn 1876
	PV+ (n = 36)<br>Yes (p = 0.24)		Nkx2.1+ vs FoxP2+	PV+<br>Mdn 1685
	FoxP2+ (n = 21)<br>No (p = 0.007))		PV+ vs FoxP2+	FoxP2+<br>Mdn 1317
**Bifurcation count**	Nkx2.1+ (n = 24)<br>Yes (p = 0.21)	F (2, 78) = 0.397; p = 0.67	Nkx2.1+ vs PV+	Nkx2.1+<br>M 10.88 ± 0.99
	PV+ (n = 36)<br>No (p = 0.0007)		Nkx2.1+ vs FoxP2+	PV+<br>M 9.69 ± 1.097
	FoxP2+ (n = 21)<br>Yes (p = 0.079)		PV+ vs FoxP2+	FoxP2+<br>M 9.62 ± 0.94

**Maximal dendritic length (µm)**	Nkx2.1+ (n = 24)<br>Yes (p = 0.2)<br>PV+ (n = 36)<br>Yes (p = 0.1)<br>FoxP2+ (n = 21)<br>Yes (p = 0.19)	F (2, 78) = 3.93; p = 0.02	Nkx2.1+ vs PV+<br>P = 0.66<br>Nkx2.1+ vs FoxP2+<br>P = 0.17<br>PV+ vs FoxP2+<br>P = 0.018	Nkx2.1+<br>M 339.3 ± 14.97<br>PV+<br>M 358.9 ± 16.64<br>FoxP2+<br>M 293.6 ± 14.66
**Total surface area (μm^2)**	Nkx2.1+ (n = 24)<br>Yes (p = 0.075)<br>PV+ (n = 36)<br>No (p = 0.026)<br>FoxP2+ (n = 21)<br>No (p = 0.007)	H (2) = 8.032; p = 0.018	Nkx2.1+ vs PV+<br>P = 0.49<br>Nkx2.1+ vs FoxP2+<br>P = 0.014<br>PV+ vs FoxP2+<br>P = 0.24	Nkx2.1+<br>Mdn 8390<br>PV+<br>Mdn 6823<br>FoxP2+<br>Mdn 4677
**Soma area (μm^2)**	Nkx2.1+ (n = 24)<br>Yes (p = 0.13)<br>PV+ (n = 36)<br>Yes (p = 0.16)<br>FoxP2+ (n = 20)<br>Yes (p = 0.21)	F (2, 78) = 13.58; p < 0.0001	Nkx2.1+ vs PV+<br>P = 0.29<br>Nkx2.1+ vs FoxP2+<br>P = 0.0026<br>PV+ vs FoxP2+<br>P < 0.0001	Nkx2.1+<br>M 588.9 ± 33.32<br>PV+<br>M 652.5 ± 30.1<br>FoxP2+<br>M 422.5 ± 25.97
**Primary dendrite count**	Nkx2.1+ (n = 25)<br>No (p = 0.035)<br>PV+ (n = 36)<br>Yes (p = 0.002)<br>FoxP2+ (n = 20)<br>Yes (p = 0.05)	H (2) = 0.57; p = 0.75	-<br>-<br>-<br>-<br>-<br>-	Nkx2.1+<br>Mdn 3<br>PV+<br>Mdn 3<br>FoxP2+<br>Mdn 3
**Scholl analysis**	Nkx2.1+ (n = 24)<br>No (p = 0.014)<br>PV+ (n = 36)<br>No (p = 0.005)<br>FoxP2+ (n = 21)<br>No (p = 0.005)	Two-way ANOVA: Group comparison F (2, 78) = 0.74; p = 0.48	-<br>-<br>-<br>-<br>-<br>-	
**Dendritic diameter (µm) 1st order**	Nkx2.1+ (n = 21)<br>No (p = 0.03)<br>PV+ (n = 11)<br>No (p = 0.027)<br>FoxP2+ (n = 13)<br>Yes (p = 0.58)	H (2) = 10.73; p = 0.0047	Nkx2.1+ vs PV+<br>P > 0.999<br>Nkx2.1+ vs FoxP2+<br>P = 0.0077<br>PV+ vs FoxP2+<br>P = 0.025	Nkx2.1+<br>Mdn 1.66<br>PV+<br>Mdn 1.56<br>FoxP2+<br>Mdn 1.39
**Dendritic diameter (µm) 2nd order**	Nkx2.1+ (n = 118)<br>No (p < 0.0001)<br>PV+ (n = 63)<br>No (p < 0.0001)<br>FoxP2+ (n = 74)<br>No (p < 0.0001)	H (2) = 22.97; p < 0.0001	Nkx2.1+ vs PV+<br>P = 0.13<br>Nkx2.1+ vs FoxP2+<br>P < 0.0001<br>PV+ vs FoxP2+<br>P = 0.06	Nkx2.1+<br>Mdn 1.20<br>PV+<br>Mdn 1.07<br>FoxP2+<br>Mdn 0.92
**Dendritic diameter (µm) >2nd order**	Nkx2.1+ (n = 327)<br>No (p = 0.0001)<br>PV+ (n = 171)<br>No (p = 0.0061)<br>FoxP2+ (n = 173)<br>No (p < 0.0001)	H (2) = 26.47; p < 0.0001	Nkx2.1+ vs PV+<br>P = 0.154<br>Nkx2.1+ vs FoxP2+<br>P < 0.0001<br>PV+ vs FoxP2+<br>P = 0.016	Nkx2.1+<br>Mdn 1.02<br>PV+<br>Mdn 0.92<br>FoxP2+<br>Mdn 0.83
